# Arginase 2 negatively regulates sorafenib-induced cell death by mediating ferroptosis in melanoma: The role of Arginase 2 in sorafenib-induced ferroptosis in melanoma

**DOI:** 10.3724/abbs.2022166

**Published:** 2022-11-09

**Authors:** Yi Yu, Yuanyuan Ren, Caihua Wang, Zhuozhuo Li, Fanglin Niu, Zi Li, Qiang Ye, Jiangxia Wang, Yuan Yan, Ping Liu, Lu Qian, Yuyan Xiong

**Affiliations:** 1 Xi’an Key Laboratory of Cardiovascular and Cerebrovascular Diseases Xi’an No.3 Hospital Faculty of Life Sciences and Medicine Northwest University Xi’an 710018 China; 2 Key Laboratory of Resource Biology and Biotechnology in Western China Ministry of Education School of Medicine Northwest University Xi’an 710069 China; 3 Department of Endocrinology Xi’an No.3 Hospital the Affiliated Hospital of Northwest University Northwest University Xi’an 710069 China

**Keywords:** Arginase 2, sorafenib, ferroptosis, lipid peroxidation, melanoma

## Abstract

Ferroptosis, a newly defined and iron-dependent cell death, morphologically and biochemically differs from other cell deaths. Melanoma is a serious type of skin cancer, and the poor efficacy of current therapies causes a major increase in mortality. Sorafenib, a multiple kinase inhibitor, has been evaluated in clinical phase trials of melanoma patients, which shows modest efficacy. Emerging evidence has demonstrated that arginase 2 (Arg2), type 2 of arginase, is elevated in various types of cancers including melanoma. To investigate the role and underlying mechanism of Arg2 in sorafenib-induced ferroptosis in melanoma, reverse transcriptase-quantitative polymerase chain reaction, western blot analysis, adenovirus and lentivirus transduction, and *in vivo* tumor homograft model experiments were conducted. In this study, we show that sorafenib treatment leads to melanoma cell death and a decrease in Arg2 at both the mRNA and protein levels. Knockdown of *Arg2* increases lipid peroxidation, which contributes to ferroptosis, and decreases the phosphorylation of Akt. In contrast, overexpression of Arg2 rescues sorafenib-induced ferroptosis, which is prevented by an Akt inhibitor. In addition, genetic and pharmacological suppression of Arg2 is able to ameliorate the anticancer activity of sorafenib in melanoma cells *in vitro* and in tumor homograft models. We also show that Arg2 suppresses ferroptosis by activating the Akt/GPX4 signaling pathway, negatively regulating sorafenib-induced cell death in melanoma cells. Our study not only uncovers a novel mechanism of ferroptosis in melanoma but also provides a new strategy for the clinical applications of sorafenib in melanoma treatment.

## Introduction

Cell death plays a vital role in various physiological processes of embryonic development, tissue homeostasis and immunity in mammals [1]. Dysregulation of this process is associated with diverse diseases, including cancer, cardiovascular diseases, immunological and developmental disorders and neurodegeneration [2]. In 2012, ferroptosis, a novel death type, was first termed by Stockwell’s Lab to define RAS-selective lethal small molecule erastin-induced human fibrosarcoma cell death [3], which is iron-dependent and morphologically and biochemically distinct from autophagy-dependent cell death, necrosis, apoptosis, and pyroptosis [4]. Accumulating evidence indicates that ferroptosis tightly mediates oxidative stress and inflammatory reactions, which are associated with a variety of diseases, including cancer, kidney injury, cardiovascular diseases and Huntington disease [5].

Biochemically, drained intracellular glutathione (GSH) and inactivated glutathione peroxidase 4 (GPX4) lead to overproduction of lipid peroxides that cannot be scavenged by the GPX4-catalyzed reaction, resulting in overwhelming lipid-reactive oxygen species (lipid-ROS) production, which finally induces ferroptosis [6]. The ferroptosis-inducing agent erastin is capable of draining GSH by inhibiting cystine/glutamate antiporter (system X _c_^‒^), resulting in an increase in lipid ROS and eventual ferroptosis [ 7, 8] . Direct GPX4 inhibitors, such as RSL3, significantly evoke the generation of lipid hydroperoxides and toxic aldehydes, such as malondialdehydes (MDAs), which by crosslinking might inactivate essential cellular proteins to trigger ferroptosis [6]. Moreover, accumulated intracellular iron is another key to executing ferroptosis through the facilitation of the Fenton reaction producing lipid hydroperoxides [9]. Therefore, iron regulation and lipid peroxidation generation signaling are recognized as core regulators of ferroptosis.

Melanoma is a highly malignant type of skin cancer, and its incidence has increased annually over the past decades [10]. The limited efficacy of current treatments, including single or combined agents, results in an increase in mortality from melanoma [11]. Melanogenesis, a biological and biochemical process producing melanin in melanoma cells, plays a vital role in the regulation of melanoma behavior as well as progression [ 12‒ 14] . During the process of melanogenesis, melanoma cells consume cysteine, which may cause a reduction in GSH production, leading to weakened capacity of the cellular antioxidative defense and accumulation of ROS, which in turn exacerbates melanogenesis [ 15, 16] . Moreover, iron, as a crucial regulator of ferroptosis, has been found to promote melanogenesis in retinal pigment epithelial cells [17]. Therefore, ferroptosis is quite likely to be implicated in the pathogenesis and progression of melanoma. Sorafenib, a multiple kinase inhibitor and Food and Drug Administration (FDA)-approved compound, has been evaluated in clinical phase IV trials of melanoma patients, which showed that sorafenib had modest efficacy in melanoma [18]. Therefore, new strategies for sorafenib in melanoma treatment are necessary to improve its efficacy. Emerging studies have revealed that sorafenib promotes ferroptotic cell death in different kinds of cancer cell lines, such as hepatocellular carcinoma cells [19], human kidney cancer cells and non‑small cell lung cancer [ 19, 20] . However, whether sorafenib also induces ferroptotic cell death and the underlying molecular mechanisms in melanoma remain elusive.

Arginase 2 (Arg2), type 2 of arginase metabolizing L-arginine to L-ornithine and urea, is abundantly expressed in extrahepatic tissues or cells [21]. L-Ornithine is the precursor for synthesizing polyamines that are essential in cell growth and protein biosynthesis [22]. Researchers have reported that upregulated Arg2 expression is implicated in tumors with high grade and patient prognosis prediction [ 23, 24] and that the suppression of arginase inhibits cancer cell growth [ 25, 26] . Our previous studies demonstrated that Arg2 is able to promote melanoma cell proliferation through polyamine and metastasis-related processes independent of its enzymatic activity [27]. Although a strong correlation between Arg2 and cancer has been reported in both basic and clinical research, the role of Arg2 in the antitumour effects of sorafenib in melanoma is still not elucidated. In the present study, we demonstrate that sorafenib treatment leads to the downregulation of Arg2 at the mRNA and protein expression levels, which suppresses ferroptosis by activating the Akt/GPX4 signaling pathway in mouse melanoma cells. Moreover, genetic depletion of Arg2 expression and pharmacological suppression of Arg2 activity *in vitro* and *in vivo* ameliorate the antitumour activity of sorafenib in melanoma cells. In summary, our study not only reveals a novel mechanism of sorafenib-induced ferroptosis but also provides a new strategy for the clinical application of sorafenib in melanoma therapy.

## Materials and Methods

### Reagents

Rabbit antibodies against Arginase 2, p-Akt and GPX4 were obtained from Cell Signaling Technology (Boston, USA); antibody for Akt detection was purchased from BD Biosciences (San Jose, USA); anti-actin antibody was purchased from Origene (Rockville, USA). IRDye 800CW goat anti-rabbit IgG (H+L) secondary antibody was purchased from LI-COR Biotechnology (Lincoln, USA), and goat anti-mouse IgG (H+L) secondary antibody was purchased from Thermo Fisher Scientific (Waltham, USA). All the cell culture media and materials were purchased from Gibco (Waltham, USA) or Sigma‒Aldrich (St Louis, USA).

### Cell culture and adenoviral/lentiviral transduction

The mouse melanoma B16F10 cell line used in our study was purchased from ATCC (Manassas, USA) and seeded in 100-cm peri dishes containing high glucose-DMEM (Sigma‒Aldrich) supplemented with 10% fetal bovine serum and 1% penicillin‒streptomycin antibiotic solution. The human melanoma cell line ME276 derived from the lymph nodes of two different patients with melanoma metastasis was cultured in Roswell Park Memorial Institute (RPMI) 1640 medium containing 10% fetal calf serum (FCS) [27]. Adenovirus/lentivirus transduction for gene overexpression or silencing was performed as previously described [28].

### Recombinant adenovirus production

The expression plasmid encoding murine arginase 2 (pCMV6-Arg2) was obtained from OriGene Technologies (Nunningen, Switzerland). Recombinant adenovirus (rAd) expressing Arg2 was generated with Gateway Recombination Cloning Technology (Thermo Fisher Scientific) according to the manuals. The empty rAd/CMV used as the control rAd was also from Thermo Fisher Scientific.

### Lentivirus generation

HEK293T cells were transfected with pLKO.1 plasmids with targeted shRNA sequences for gene knockdown or pLJM1 with an Arg2 gene insert for gene overexpression together with psPAX2 and pMD2.G plasmids using Lipo 6000 Transfection Reagents (Beyotime Biotechnology) to generate lentivirus particles. After transfection, HEK293T cells were cultured for 48 h, and then the supernatant was centrifuged at 1500 *g* for 5 min to collect viral particles. The sequences of the sense strand targeting mouse GPX4 and Arg2 are as follows: *GPX4* shRNA: 5′-GTCGATCTGCATGCCCGATAT-3′; *Arg2* shRNA: 5′-CACAAGATGATCCCTACAATA-3′; and scramble shRNA (5′-CCTAAGGTTAAGTCGCCCTCG-3′) was a gift from David Sabatini’s Lab deposited in Addgene (Cambridge, Watertown, USA) [29].

### Mouse model of subcutaneous melanoma

To form murine subcutaneous melanoma tumors, various groups of 1×10 ^6^/mouse B16F10 cells were injected subcutaneously into the right flanks of female C57BL/6 mice aged 6 to 12 weeks. Tumor growth and volume were monitored daily and estimated according to the modified ellipsoidal formula based on calliper measurements (tumor volume=(length×width ^2^)/2) [30]. After 7 days of injection, mice were allocated randomly into several groups and treated with sorafenib or sorafenib plus the indicated inhibitors BEC [31] or LY29002 [32] for 3 weeks. Following sorafenib treatment, the formed tumor tissues were isolated. All animal experiments were approved by the Animal Ethics Committee of Northwest University and performed in accordance with the Association for Assessment and Accreditation of Laboratory Animal Care guidelines.

### Quantitative real-time reverse transcription PCR (qRT-PCR)

RNA was extracted by using TRIzol reagent, and mRNA expressions were measured by 2-step qRT-PCR using SYBR (Promega, Madison, USA) as described previously [21]. *GAPDH* was used for normalization. The sequences of the primers used are as follows: *Arg1*-Forward: 5′-CTCCAAGCCAAAGTCCTTAGAG-3′, *Arg1*-Reverse: 5′-AGGAGCTGTCATTAGGGACATC-3′; *Arg2*-Forward: 5′-TCCTGGATCAAACCTTGCCT-3′, *Arg2*-Reverse: 5′-CCTTTTGCCAATCAGCCGAT-3′; *GPX4*-Forward: 5′-GATGGAGCCCATTCCTGAACC-3′, *GPX4*-Reverse: 5′-CCCTGTACTTATCCAGGCAGA-3′; *SLC7A11*-Forward: 5′-TCCTGCTTTGGCTCCATGAACG-3′, *SLC7A11*-Reverse: 5′-AGAGGAGTGTGCTTGCGGACAT-3′; *SLC3A2*-Forward: 5′-CCAGAAGGATGATGTCGCTCAG-3′, *SLC3A2*-Reverse: 5′-GAGTAAGGTCCAGAATGACACGG-3′; *GAPDH*-Forward: 5′-ACCCAGAAGACTGTGGATGG-3′, *GAPDH*-Reverse: 5′-ACACATTGGGGGTAGGAACA-3′.

### Western blot analysis

Protein extraction from cells, sample preparation, SDS‒PAGE electrophoresis and transfer were performed as previously described [21]. The resultant membrane was first blocked with 5% skimmed milk, then incubated with primary antibody at room temperature for 2 h or at 4°C overnight, and finally probed by corresponding secondary antibody. Signals were detected with an Odyssey Infrared Imaging System (LI-COR Biosciences, Bad Homburg, Germany) and quantified using ImageJ software (NIH, Bethesda, USA).

### Cell viability assay

Cell viability was determined by using the Cell Counting Kit-8 (CCK-8) (Beyotime Biotechnology, Shanghai, China) according to the manufacturer’s instructions. Absorbance at 450 nm was measured with a microplate reader. The cell viability of each group was calculated as the percentage of absorbance of treated cells compared with that of cells in the control group.

### Lipid peroxidation detection

The lipid peroxidation assay in cell lysates was evaluated by using the Colorimetric/Fluorometric Lipid Peroxidation Assay Kit (Abcam, Cambridge, UK) to determine the level of malondialdehyde (MDA) according to the manufacturer’s instructions.

### Iron determination

Cellular ferrous iron levels were examined by using an Abcam iron assay kit (Abcam) according to the manufacturer’s manuals. Briefly, cells were digested with trypsin-EDTA and collected by centrifugation. After homogenization in iron assay buffer on ice and centrifugation, the iron probe was added to each supernatant and incubated for 1 h at 37°C in the dark. Then, the absorbance at 450 nm was immediately read.

### Colony formation assay

Cultured B16F10 cells with 80% confluence were digested with 0.25% Trypsin-EDTA digestion solution. Following cell number counting, approximately 300 cells were seeded in a 6-cm plate. After 24 h of incubation, the cells were treated with sorafenib for another 24 h and then cultured in fresh medium for 4–8 days. After the treatment, the cells were fixed with 4% paraformaldehyde, rinsed with distilled water and stained with freshly prepared 0.2% crystal violet for 10 min at ambient temperature. The colonies (at least consisting of 50 cells) were counted under a microscope. The colony formation rate was calculated according to the following formula: (numbers of colonies/numbers of seeded cells)×100%.

### Statistical analysis

Data are presented as the mean±SEM. Unpaired Student’s *t*-test or ANOVA with Bonferroni post-test was employed for statistical analysis using GraphPad Prism 8.0. It is considered statistically significant when the *P* value is less than 0.05.

## Results

### Sorafenib induces ferroptosis and suppresses Arg2 expression in B16F10 cells

**Figure 1:**
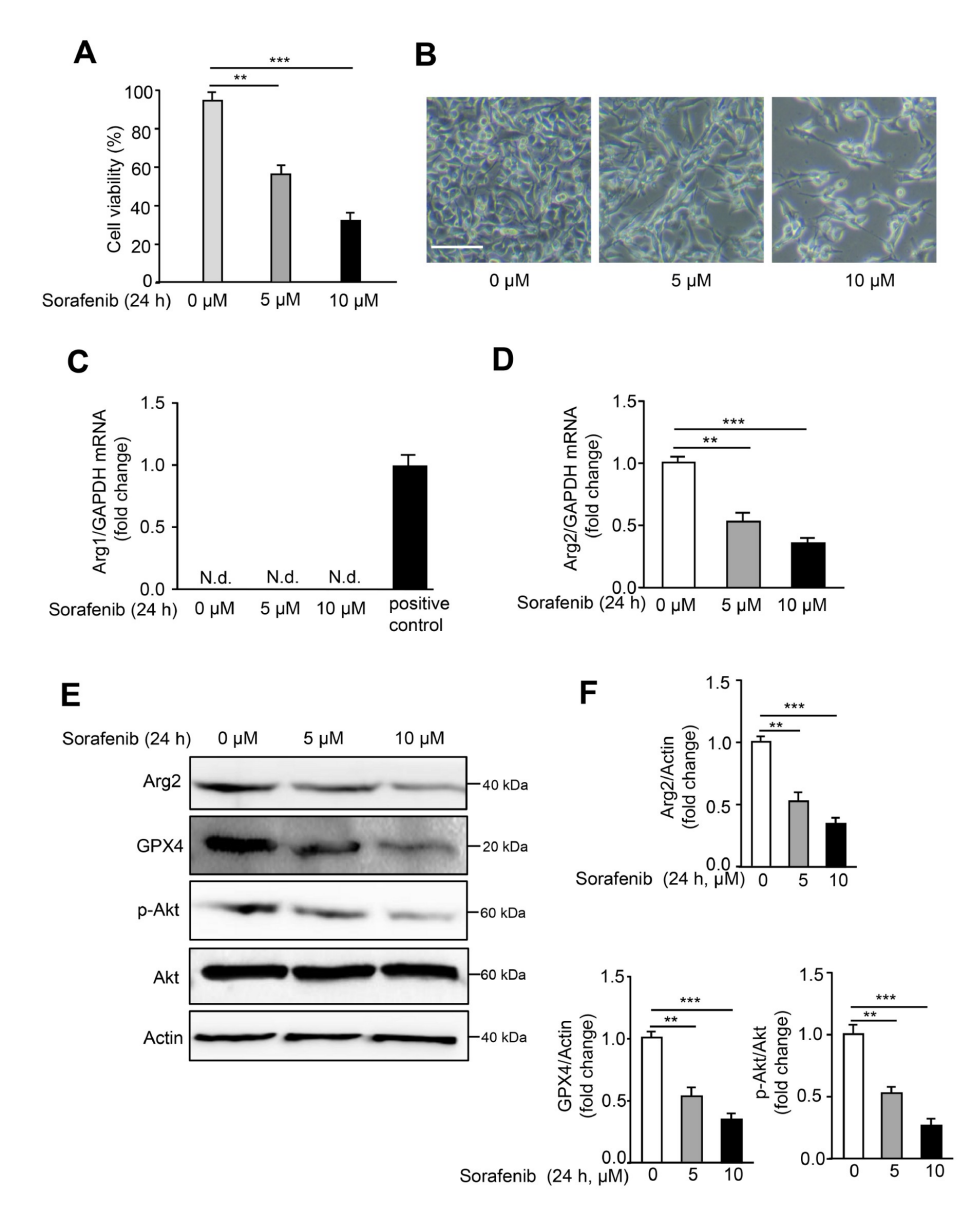
Figure 1 Sorafenib promotes ferroptosis and suppresses Arg2 expression in B16F10 cells (A) B16F10 cells were treated with DMSO (0 μM) or sorafenib (5 or 10 μM) for 24 h, and cell viability was assayed by CCK8 assay. (B) Representative images of sorafenib-induced cell death. (C,D) The mRNA levels of Arg1 and Arg2 were determined by qRT-PCR. Mouse liver tissue was used as a positive control for Arg1. N.d.: not detectable. (E) Western blot analysis of the expression levels of Arg2, p-Akt, Akt, and GPX4. Actin was used as a loading control. (F) The quantification of protein expression levels in (E). Scale bars=100 μm ( n=3). ** P<0.01, *** P<0.001.

Sorafenib is a classical small-molecule inducer of ferroptosis and has been assessed in phase I, II and III clinical trials in various types of cancers, such as hepatocellular carcinoma (HCC), non-small cell lung cancer, colorectal cancer, and melanoma [10]. As shown in Figure 1A,B, the cell viability of the murine melanoma cell line B16F10 significantly dropped with increasing sorafenib concentration, and treatment with 5 μM for 24 h induced nearly 50% cell death. The decrease in the GPX4 expression level, one essential hallmark of ferroptosis, indicated ferroptosis promoted by sorafenib in B16F10 cells. To examine the effects of sorafenib on arginase expression, Arg1 and Arg2 protein and mRNA expression levels were analyzed by western blot analysis and qPCR. Intriguingly, both the protein and mRNA expression levels of Arg2 but not arginase 1 (Arg1) were significantly inhibited with sorafenib treatment, and this trend was more prominent with increasing sorafenib concentration ( Figure 1C,D). Arg1 was hardly detected in B16F10 cells with or without sorafenib. In addition, Arg2 expression was also examined in response to other ferroptosis inducers, erastin and RSL, which function as inhibitors of system X _c_^–^ and GPX4, respectively. Further experiments showed that erastin but not RSL significantly suppressed Arg2 expression in response to another ferroptosis-inducing agent ( Supplementary Figure S1). Moreover, remarkable attenuation of Akt phosphorylation (Ser473) accompanied by an Arg2 decrease was observed under sorafenib treatment ( Figure 1E,F). In the human melanoma cell line ME276, consistent results were also obtained ( Supplementary Figure S2). These data indicated that sorafenib induced ferroptosis in mouse melanoma cells, possibly by specifically suppressing Arg2 expression.

### Arg2 overexpression rescues sorafenib-induced ferroptosis through upregulation of GPX4 and Akt

**Figure 2:**
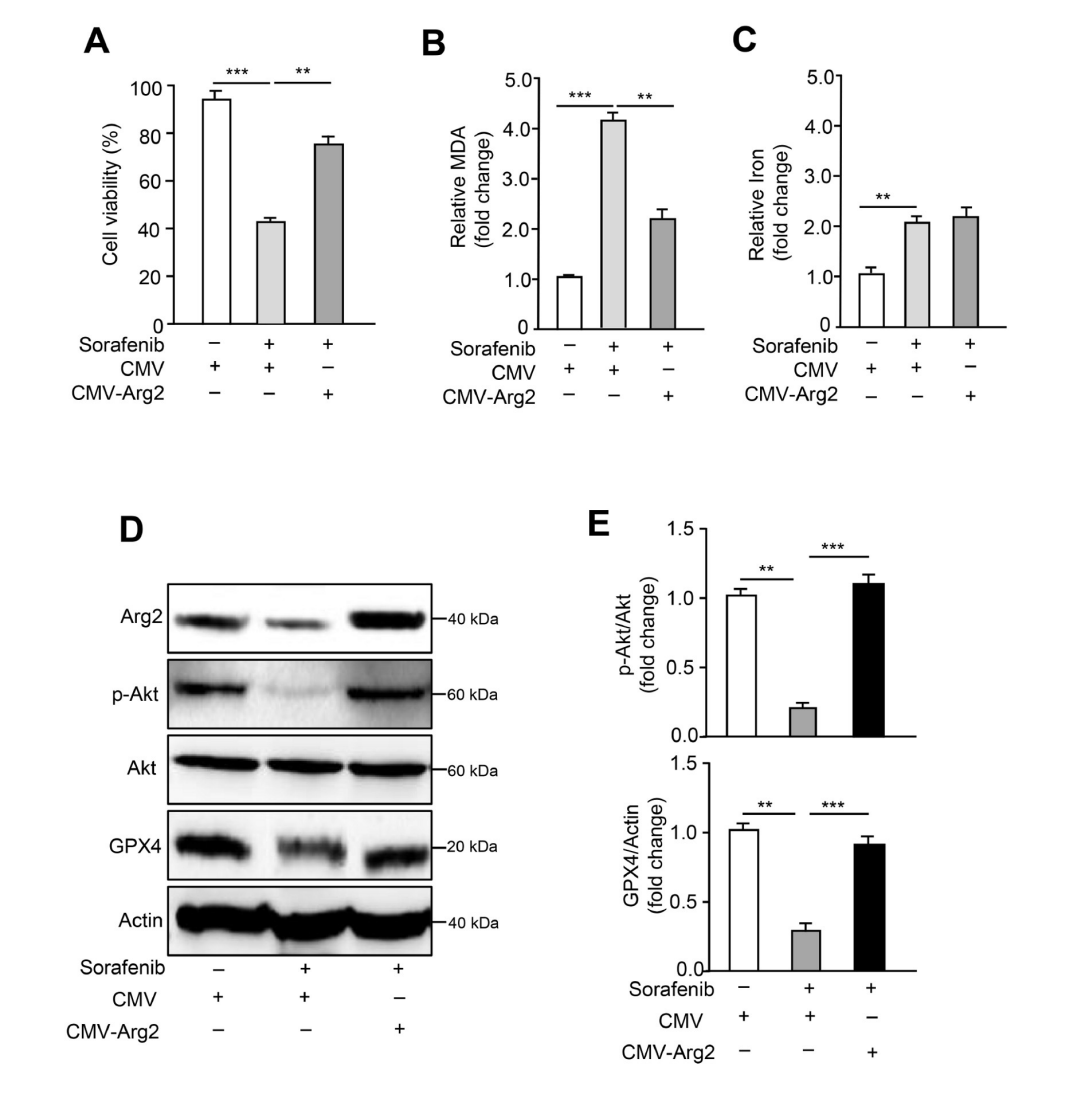
Figure 2 Arg2 overexpression rescues ferroptosis and downregulates GPX4 expression and Akt phosphorylation induced by sorafenib B16F10 cells were transduced with empty rAd/CMV (control) or rAd/CMV-Arg2. Forty-eight hours after transduction, cells were treated with DMSO or sorafenib (5 μM) for 24 h. For each group, (A) cell viability, (B) lipid peroxidation (MDA assay), and (C) iron levels were assayed. (D) Western blot analysis of the protein expression levels of Arg2, p-Akt, Akt, and GPX4. Actin was used as a loading control. (E) The quantification of protein expression levels in (D). n=3. ** P<0.01, *** P<0.001.

To determine the role of Arg2 in sorafenib-induced ferroptosis, adenovirus-mediated ectopic overexpression of Arg2 in sorafenib-treated B16F10 cells was performed. It was found that overexpression of Arg2 was able to reverse sorafenib-induced ferroptotic cell death and enhance cell viability ( Figure 2A). Ferroptosis, a regulated form of cell death, can be triggered by the accumulation of lipid peroxidation, which can be scavenged by GPX4 [1]. Since the final reaction of polyunsaturated fatty acid peroxidation produces malondialdehyde (MDA), it is considered a lipid peroxidation marker [33]. We further investigated whether Arg2 is able to mediate sorafenib-induced lipid peroxidation. Indeed, lipid peroxidation (MDA) assay revealed that sorafenib significantly evoked MDA generation, and this effect was prevented by Arg2 overexpression ( Figure 2B). Ferrous iron (Fe ^2+^) is another key to triggering ferroptosis through the Fenton reaction to generate reactive oxygen species (ROS) and lipids [4]. However, overexpression of Arg2 did not significantly influence the levels of Fe ^2+^ following sorafenib treatment ( Figure 2C), suggesting that Arg2-mediated lipid peroxidation is independent of Fe ^2+^. Western blot analysis showed that the decrease in GPX4 and p-Akt induced by sorafenib treatment was also reversed by overexpression of Arg2 ( Figure 2D,E). In addition, Arg2 overexpression was not able to affect the mRNA expression levels of SLC7A11 and SLC3A2, which are core subunits of the X _c_^−^ system ( Supplementary Figure S3A,B). These data demonstrate that Arg2 may inhibit ferroptosis by activating the GPX4 and Akt signaling pathways to block lipid peroxidation, contributing to the mechanisms of sorafenib-induced ferroptotic cell death in B16F10 cells through the inhibition of Arg2 expression.

### The Arg2-Akt-GPX4 cascade governs sorafenib-induced ferroptosis

**Figure 3:**
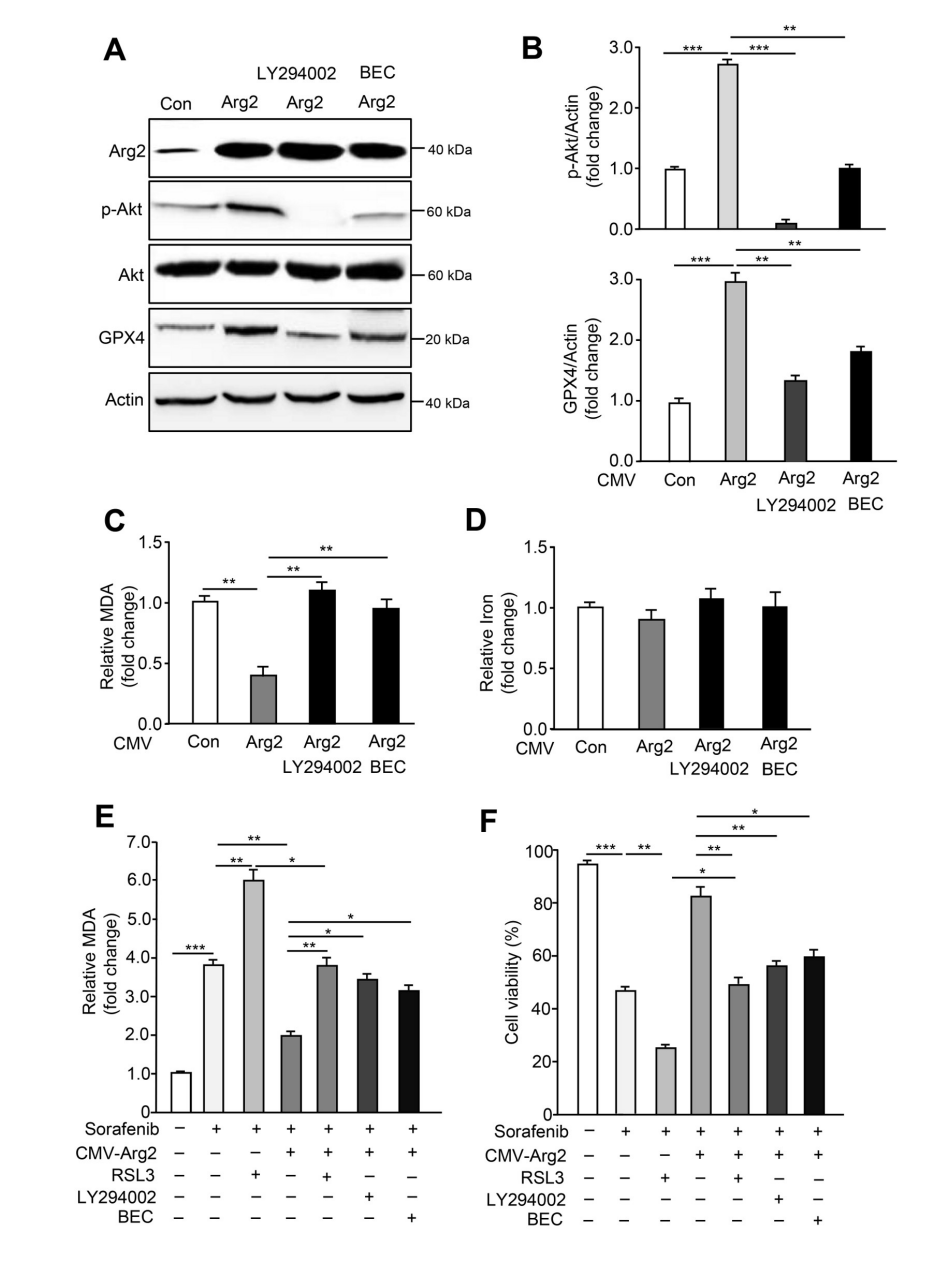
Figure 3 Arg2 promotes lipid peroxidation by activating Akt phosphorylation B16F10 cell transduction was performed with empty rAd/CMV (control) or rAd/CMV-Arg2. Forty-eight hours after transduction, the cells were starved with serum-free medium and treated with LY294002 (50 μM) or BEC (200 μM) for 24 h. (A) Western blot analysis of the protein expression levels of Arg2, p-Akt, Akt, and GPX4. Actin was used as a loading control. (B) The quantification of protein expression levels in (A). (C) Lipid peroxidation (MDA assay) and (D) iron levels were determined. Forty-eight hours after transduction with empty rAd/CMV (control) or rAd/CMV-Arg2, B16F10 cells were treated with sorafenib (5 μM) plus RSL3 (0.5 μM)or LY294002 (50 μM) or BEC (200 μM) for 24 h. For each group, (E,F) lipid peroxidation (MDA assay) and cell viability were evaluated. n=3. * P<0.05, ** P<0.01, *** P<0.001.

To further explore the potential mechanisms by which Arg2 inhibits ferroptosis, we overexpressed Arg2 by adenovirus in B16F10 cells and examined the p-Akt and GPX4 signaling cascades. As shown in Figure 3A, the overexpression of Arg2 was verified by western blot analysis, and both p-Akt and GPX4 were significantly upregulated upon Arg2 overexpression. Moreover, inhibition of Akt by the phosphoinositide 3-kinase (PI3K) inhibitor LY294002 blocked the increase in GPX4 upon Arg2 overexpression ( Figure 3A,B), indicating that Arg2 promotes GPX4 upregulation by activating Akt. S-(2-boronoethyl)-L-cysteine (BEC), a competitive arginase inhibitor that strikingly suppresses Arg2 enzymatic activity ( Supplementary Figure S4), also exhibited consistent results with LY294002 ( Figure 3A,B). Next, we examined the effects of LY294004 and BEC on Arg2-mediated lipid peroxidation. Figure 3C shows that overexpressing Arg2 suppressed lipid peroxidation, and the blockade of Akt phosphorylation by LY294002 and arginase activity by BEC were capable of restoring Arg2-induced lipid peroxidation suppression. Iron assay indicated that Arg2 overexpression with or without LY294002 and BEC treatment did not affect the level of Fe ^2+^ ( Figure 3D). To further confirm that sorafenib induces ferroptosis in B16F10 cells through the Arg2-Akt-GPX4 cascade, cell viability and lipid peroxidation were determined in cells treated with various inhibitors, including RSL3 (GPX4 inhibitor), LY294004 and BEC, following sorafenib treatment and Arg2 overexpression. As expected, overexpression of Arg2 rescued sorafenib-induced cell death and inhibited MDA production. However, compared to sorafenib or sorafenib plus RSL3 treatment, RSL3, LY294004 and BEC treatment prevented the protective effect of Arg2 on sorafenib-induced ferroptosis ( Figure 3E,F). Genetic deletion of *GPX4* also showed consistent results with RSL3 treatment ( Supplementary Figure S5). Collectively, these results suggest that sorafenib induces the downregulation of Arg2 expression, which further suppresses the Akt-GPX4 cascade, contributing to the elevation of lipid peroxidation production and ferroptosis occurrence.

### Suppression of Arg2 enhances the sensitivity to sorafenib-induced cell death

**Figure 4:**
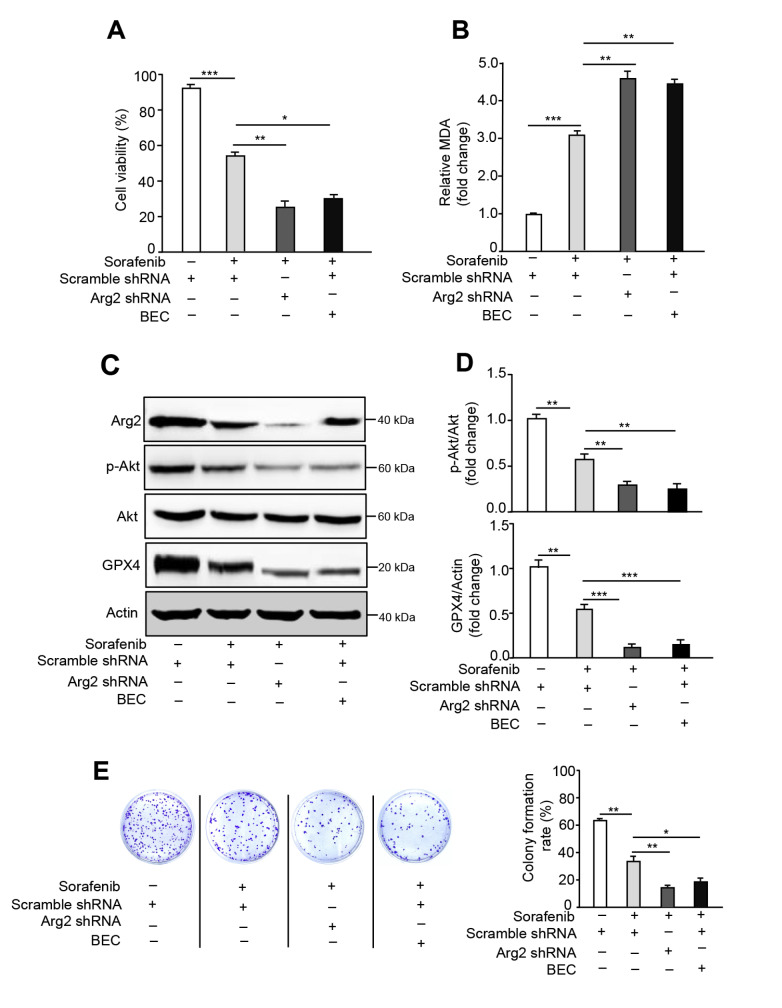
Figure 4 Inhibition of Arg2 exacerbates sorafenib-induced ferroptosis B16F10 cell transduction was performed with scramble shRNA (control) or Arg2 shRNA lentivirus. Forty-eight hours after transduction, cells were treated with sorafenib (5 μM) plus BEC (200 μM) or left untreated for 24 h. (A, B) For each group, cell viability and lipid peroxidation were measured. (C) Western blot analysis of the protein expression levels of Arg2, p-Akt, Akt, and GPX4. Actin was used as a loading control. (D) The quantification of protein expression levels in (C). (E) Colony formation assay of each group. n=3. * P<0.05, ** P<0.01, *** P<0.001.

Sorafenib resistance impedes the clinical application of this drug in cancer treatment; therefore, enhancing the sensitivity of sorafenib-induced cancer cell death is a crucial strategy to facilitate sorafenib therapy [ 34, 35] . To investigate whether knockdown of Arg2 or arginase activity inhibition ameliorates the sensitivity of sorafenib-induced cell death, either Arg2 shRNA lentivirus or BEC treatment was employed in B16F10 cells. Indeed, depletion of Arg2 or arginase suppression significantly exacerbated the cell death, as revealed by cell viability assay, as well as lipid peroxidation indicated by the MDA assay ( Figure 4A,B). Western blot analysis showed that silencing *Arg2* or suppressing its activity further inhibited the sorafenib-induced decrease in p-Akt and GPX4 expression ( Figure 4C,D). Additionally, the colony formation assay also showed that inhibition of Arg2 expression or activity was more prominent ( Figure 4E). These results suggest that Arg2 expression depletion or activity suppression is capable of enhancing sorafenib antitumour sensitivity in melanoma.

### Targeting Arg2 strengthens the antitumour activity of sorafenib *in vivo*

**Figure 5:**
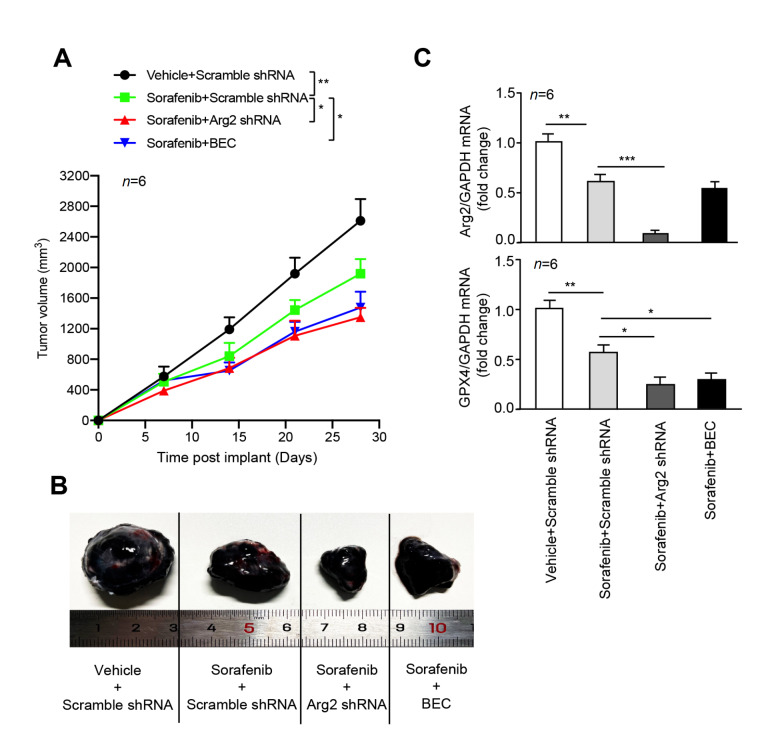
Figure 5 Depletion of Arg2 enhances sorafenib sensitivity *in vivo* C57BL/6 mice were implanted subcutaneously with the indicated 1×10 6 B16F10 cells/mouse and treated intraperitoneally with only sorafenib (three times a week, 10 mg/kg) or sorafenib plus arginase inhibitor BEC (three times a week, 2.3 mg/kg intraperitoneally) at day 7 for 3 weeks ( n=6 per group). (A) Tumor volume calculation. (B) At day 28, the tumors of each group were isolated, and Arg2 and GPX4 mRNA levels in isolated tumors were determined by qRT-PCR. (C) Representative images of isolated tumors at day 28. n=6. * P<0.05, ** P<0.01, *** P<0.001.

**Figure 6:**
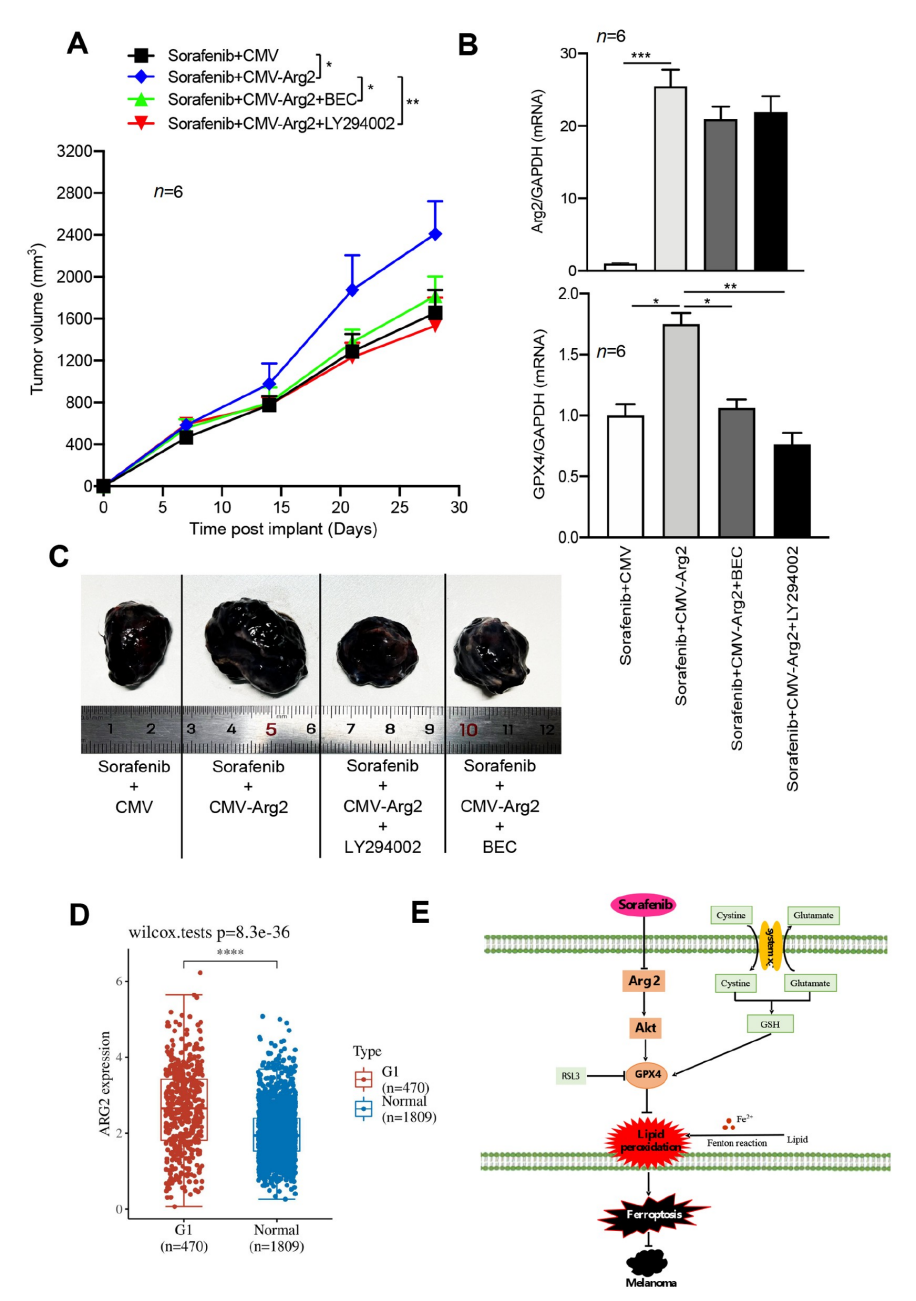
Figure 6 Hyperactive Arg2 suppresses sorafenib sensitivity *in vivo* C57BL/6 mice were injected subcutaneously with the indicated B16F10 cells at 1×10 6cells/mouse and treated intraperitoneally with only sorafenib (three times a week, 10 mg/kg) or sorafenib plus BEC (2.3 mg/kg intraperitoneally,three times a week) or LY294002 (three times a week, 100 mg/kg intraperitoneally) at day 7 for 3 weeks ( n=6 mice/group). (A) Tumor volume calculation. (B) The mRNA levels of Arg2 and GPX4 in isolated tumors at day 28 were determined by qRT-PCR. n=6. * P<0.05, ** P<0.01, *** P<0.001. (C) Representative images of isolated tumors at day 28. (D) TCGA and GTEx database data showing the significant upregulation of ARG2 mRNA in melanoma patients. (E) Schematic summary of the findings of this study.

To further validate whether knockdown of Arg2 enhances the antitumour effects of sorafenib *in vivo*, Arg2-deficient B16F10 cells were implanted into the right flanks of mice by subcutaneous injection using a homograft model. After 7 days of implantation, sorafenib and BEC were administered by intraperitoneal injection. As expected, sorafenib treatment decreased the melanoma tumor size ( Figure 5A,B). Compared to the control group, *Arg2* knockout or arginase inhibition with BEC further decreased the melanoma tumor size of sorafenib-treated mice ( Figure 5A,B). qRT-PCR analysis of isolated tumor tissues from implanted mice showed that Arg2 mRNA expression was significantly inhibited, accompanied by a decrease in GPX4 level ( Figure 5C). In contrast, overexpression of Arg2 in B16F10 cells was able to rescue the decrease in tumor volume induced by sorafenib ( Figure 6A,B). However, this rescue could be prevented by LY294002 or BEC treatment ( Figure 6A,B). The overexpression of Arg2 was confirmed by qPCR analysis of isolated tumors from implanted mice ( Figure 6C). The *in vivo* results are consistent with our previous finding that LY294002 or arginase inhibition prevents Arg2 overexpression from restoring the sorafenib-induced GPX4 decrease in B16F10 cells. These results suggest that genetic or pharmacological suppression of Arg2 expression or activity renders higher sensitivity to sorafenib by inducing ferroptosis *in vivo*. Moreover, Genotype-Tissue Expression (GTEx) and Cancer Genome Atlas (TCGA) database analyses revealed that Arg2 mRNA expression level was abnormally upregulated in melanoma patients compared to that in the normal group ( Figure 6D).

## Discussion

Upregulated Arg2 has been reported in a variety of cancers, including melanoma [27] and pancreatic cancer [23], and the suppression of arginase inhibits tumor cell growth and metastasis [ 25, 26, 36] . Clinical database analysis for melanoma shows that Arg2 is remarkably upregulated in melanoma patients compared to the normal group ( Figure 6D). In our previous study, immunofluorescence staining analysis of clinical human melanoma specimens confirmed that Arg2 expression was remarkably upregulated in melanoma patients [27], suggesting that Arg2 plays a potential role in promoting melanoma cell growth or antagonizing melanoma cell death. Clinical phase IV trials of melanoma patients treated with sorafenib showed that it had modest efficacy against melanoma [18], which restrains the therapeutic use of sorafenib for melanoma treatment. Over the past years, several studies have reported that sorafenib strongly induces ferroptosis, but not apoptosis, in HCC cells [ 19, 34, 37] . Artesunate combined with sorafenib has been shown to strengthen the induction of ferroptosis in HCC [38]. Furthermore, proteomics analysis revealed that changes in the phosphorylation of ferroptosis-related proteins occurred during sorafenib-induced ferroptosis in human HCC [39]. More recently, Zheng *et al*. [40] reported that sorafenib failed to trigger ferroptosis via the knockout or knockin of *SLC7A11* in a wide range of cancer cell lines, including the human fibrosarcoma cell line HT1080, human embryonic kidney cell line HEK293T, and human hepatoma cell line HepG2. However, SLC3A2, the other subunit of system X _c_^−^, was not investigated in this study. As such, whether sorafenib regulates ferroptosis and the underlying mechanism still requires further exploration. In this study, we also observed that sorafenib treatment resulted in Arg2 but not Arg1 downregulation at the mRNA and protein expression levels, leading to ferroptosis, and overexpression of Arg2 blocked lipid peroxidation by activating the Akt/GPX4 signaling pathway, which negatively regulates sorafenib-induced ferroptosis in murine melanoma cells. In addition, we validated that inhibition of Arg2 expression or activity is able to enhance the antimelanoma activity of sorafenib in a mouse homograft model.

Arg1 and Arg2, as two isoforms of arginase, exist in different intracellular compartments and present distinct functions. Arg1 mainly functions in the urea cycle and anti-inflammatory responses and is located primarily in the cellular cytoplasm [ 41, 42] . The second isoform, Arg2, is located in mitochondria and absent from the urea cycle and has been implicated in the mediation of endothelial cell senescence, smooth cell apoptosis, macrophage proinflammatory responses and cancer cell growth [ 22, 23, 43, 44] . In murine and human melanoma cells treated with sorafenib, ferroptosis was significantly induced, which is consistent with previous findings in HCC cells [ 45, 46] . Interestingly, with the occurrence of ferroptosis, Arg2 was specifically downregulated in melanoma cells in response to sorafenib and erastin, indicating that Arg2 may promote melanoma cell growth or inhibit ferroptosis, which is in agreement with our previous study [27]. However, RSL, an inhibitor of GPX4, was not able to influence Arg2 expression, suggesting that the inhibition of system X _c_^−^ might specifically downregulate Arg2 expression. As the inhibition of system X _c_^−^ dysregulates the exchange of extracellular l-cystine and intracellular l-glutamate across the cellular plasma membrane [47], we speculate that system X _c_^−^ mediating Arg2 expression might be related to the change in extracellular l-cystine and intracellular l-glutamate. In addition, Arg1 was barely detected in B16F10 and ME276 cells with or without sorafenib, suggesting that Arg2 is the single isoform expressed in murine melanoma. In our *in vitro* and *in vivo* studies, overexpression of Arg2 rescued sorafenib-induced ferroptosis, further validating that Arg2 may suppress ferroptosis and that sorafenib promotes B16F10 ferroptotic cell death through inhibition of Arg2. However, the mechanism by which sorafenib inhibits Arg2 expression requires further investigation. Our previous study revealed that activating p38 mitogen-activated protein kinase, a member of the MAPK family, was capable of enhancing Arg2 expression in human endothelial cells [48]; therefore, sorafenib-induced Arg2 suppression might occur through the inhibitory effects of sorafenib on the MAPK/ERK pathway in melanoma cells [49].

Currently, the mechanism of sorafenib-induced ferroptosis remains elusive. Dixon *et al.* [50] found that sorafenib suppressed the function of system X _c_^−^ mediating cystine import, triggering the endoplasmic reticulum stress response, accumulated lipid peroxidation and ferroptosis. Another study revealed that sorafenib exposure reduced the protein level of retinoblastoma (Rb), which functions in cell proliferation. The G1/S checkpoint promoted the occurrence of ferroptosis in HCC cells [51]. In our study, it is important to note that sorafenib-induced ferroptosis, GPX4 decrease and lipid peroxidation were accompanied by downregulation of Arg2 expression and Akt phosphorylation in mouse melanoma cells, which can be prevented by overexpression of Arg2. However, Arg2 overexpression was not able to influence the mRNA expression levels of SLC7A11 and SLC3A2, indicating that Arg2 modulates ferroptosis not through the regulation of the subunits of system X _c_^−^ but by governing the transport of cysteine and glutamate. Here, we found that depletion of Arg2 further exacerbated sorafenib-induced ferroptosis, GPX4 decrease, lipid peroxidation and Akt inhibition. In contrast, overexpression of Arg2 promoted the upregulation of GPX4 and phosphorylation of Akt and reduced lipid peroxidation, which was prevented by Akt and arginase inhibitors. More importantly, disruption of GPX4 by RSL3 or genetic knockdown, or inhibition of Akt by LY294004 or suppression of arginase by BEC, was able to ablate the effects that overexpression of Arg2 rescued sorafenib-induced ferroptosis. As a result, Arg2 may reduce lipid peroxidation by activating the Akt-GPX4 signaling cascade, which in turn eventually leads to the suppression of ferroptosis in mouse melanoma B16F10 cells ( Figure 6E). Whether the Arg2-Akt-GPX4 axis modulates sorafenib-induced ferroptosis in human melanoma cells requires further validation. In line with our previous study showing that upregulation of Arg2 was able to activate Akt in endothelial cells, resulting in suppression of endothelial autophagy [28], arginase inhibitor BEC treatment prevented Arg2 overexpression-induced Akt activation in melanoma cells, indicating that this process is associated with the metabolism of its substrate L-arginine. Further efforts are necessary to decipher the mechanism by which Akt upregulates GPX4, which can directly reduce toxic lipid hydroperoxide-producing lipid peroxidation [52]. Gou *et al*. [53] found that the Akt-GPX4 signaling pathway is involved in hypoxic ischemia-induced ferroptosis. In other cancer cell lines, Yi *et al*. [54] found that sustained activation of the Akt signaling pathway suppressed ferroptosis through lipogenesis that is mediated by the sterol regulatory element-binding protein. In addition to the role of Arg2 in mediating ferroptosis via the Akt-GPX4 axis, Arg2, as a mitochondrial and urea hydrolase enzyme, might also be involved in the regulation of mitochondrial lipid peroxidation and l-arginine content. Mao *et al*. [55] found that detoxification of mitochondrial lipid peroxidation by dihydroorotate dehydrogenase (DHODH) protects against ferroptosis in mitochondria. However, whether l-arginine regulates ferroptosis remains elusive. Finally, our *in vitro* and *in vivo* studies validated that targeting Arg2 exacerbated sorafenib-induced ferroptotic melanoma cell death and the antimelanoma activity of sorafenib, indicating that Arg2 expression depletion or activity inhibition may represent a novel strategy for sorafenib therapy of melanoma.

## Supporting information

Supplementary_Figures-revised_Xiong

## Supplementary Data

Supplementary data is available at *Acta Biochimica et Biophysica Sinica* online.
