# Supplementary material for: Arginase 2 negatively regulates sorafenib-induced cell death by mediating ferroptosis in melanoma: The role of Arginase 2 in sorafenib-induced ferroptosis in melanoma
Source: Acta Biochim Biophys Sin (Shanghai). 2022 Nov 9;54(11):1658–70. doi: 10.3724/abbs.2022166 (PMC9828469; doi:10.3724/abbs.2022166)
Supplement: Supplementary_Figures-revised_Xiong [file Supplementary_Figures-revised_Xiong.pdf]

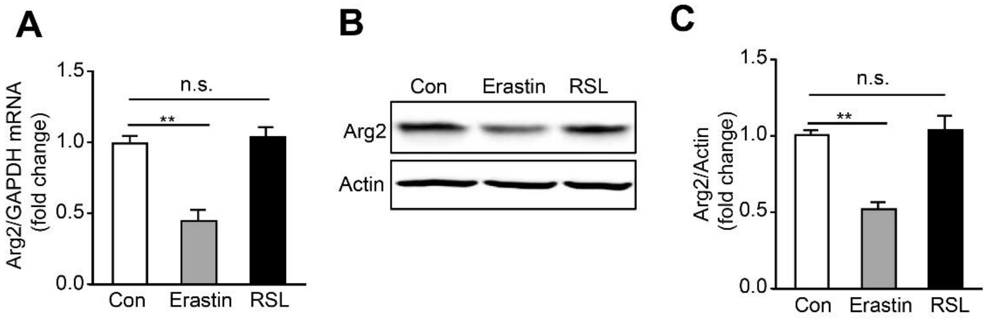

Supplementary Figure S1. Erastin but not RSL significantly suppresses the Arg2 expression B16F10 cells were treated with DMSO (Con) or erastin (10 μM) or RSL (0.5 μM) for 24 h, and subjected to (A) qRT-PCR analysis of Arg2 mRNA expression and (B) western blot analysis of Arg2 protein expression level. (C). Quantification of protein expression levels in (B). n=4. **P< 0.01, n.s.: not significant.

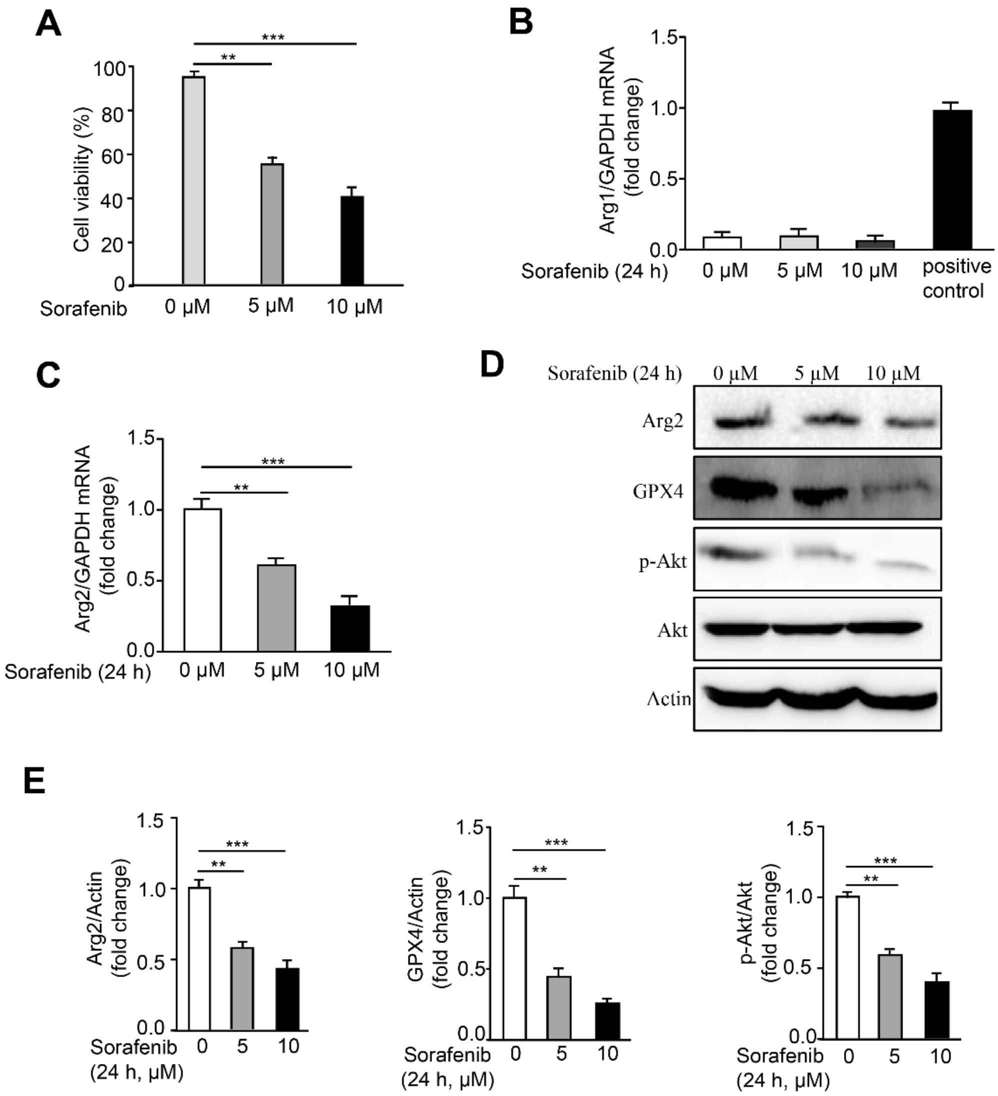

Supplementary Figure S2. Sorafenib induces ferroptosis and suppresses Arg2 expression in ME276 cells (A). Human melanoma cell line ME276 were treated with DMSO (0 μM) or sorafenib (5,10 μM) for 24 h, and cell viability was assessed by CCK8 assay. (B, C). The mRNA levels of Arg1 and Arg2 were determined by qRT-PCR. Mouse liver tissue was used as positive control for Arg1. N.d.: not detectable. (D). Western blot analysis of the expression levels of Arg2, p-Akt, Akt, and GPX4. Actin was used as loading control. (E). Quantification of protein expression levels in (E). *n*=3. ***P*< 0.01, ****P*< 0.001.

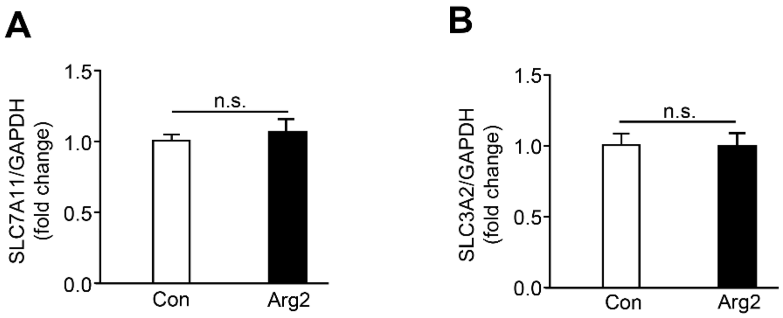

Supplementary Figure S3. Arg2 overexpression is not involved in the regulation of SLC7A11 and SLC3A2 B16F10 cells were transduced with empty rAd/CMV (control) or rAd/CMV-Arg2. Forty-eight hours after transduction, cells were subjected to qRT-PCR analysis of (A) SLC7A11 and (B) SLC3A2 mRNA expression levels. *n*=4. n.s.: not significant.

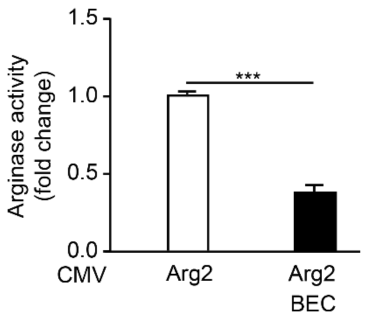

Supplementary Figure S4. BEC significantly inhibits arginase activity B16F10 cells were transduced with rAd/CMV-Arg2. Forty-eight hours after transduction, cells were treated with BEC (200 μM) for 24 h and subjected to arginase activity analysis. *n*=3. ****P*< 0.001.

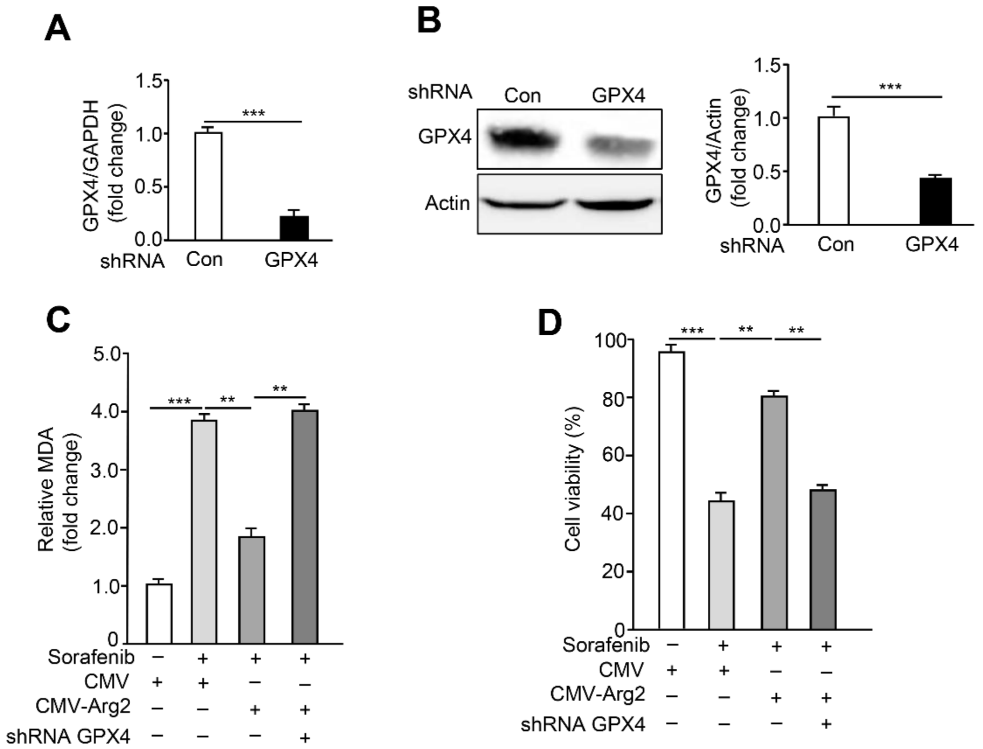

Supplementary Figure S5. Silencing GPX4 prevented the effects of Arg2 rescuing sorafenib-induced ferroptosis B16F10 cells transduction were performed with scramble shRNA (control) or GPX4 shRNA lentivirus. Post the transduction of 48 h, cells were subjected to qRT-PCR analysis of (A) GPX4 mRNA, (B) GPX4 protein expression levels, (C) the lipid peroxidation (MDA) and (D) cell viability assay. *n*=3. ***P*< 0.01, ****P*< 0.001.
